# Roles of Nitrocompounds in Inhibition of Foodborne Bacteria, Parasites, and Methane Production in Economic Animals

**DOI:** 10.3390/ani11040923

**Published:** 2021-03-24

**Authors:** Po-Yun Teng, Woo Kyun Kim

**Affiliations:** Department of Poultry Science, University of Georgia, Athens, GA 30605, USA; pyteng@uga.edu

**Keywords:** nitrocompounds, nitropropanol, nitroethanol

## Abstract

**Simple Summary:**

Supplementation of nitrocompounds in animal diets has been studied to investigate their effects on economic animals. It has been known that nitrocompounds are capable of inhibiting pathogens, parasites, methane and ammonia production. The toxicity, metabolism, and mechanisms of actions have been discussed in the review to conclude the advantages and disadvantages of application of nitrocompounds in animal production.

**Abstract:**

Nitrocompounds are derivatives of hydrocarbons, alcohols, fatty acids, and esters, consisting one or more nitro functional groups. Either natural sources of nitrocompounds or synthetic chemicals have been applied in animal diets to investigate their effects on economic animals, since conjugates of 3-nitropropanol and 3-nitropropionic acid were isolated from *Astragalus oblongifolius.* In this review, emphasis will be placed on nitrocompounds’ antimicrobial activity, toxicity, metabolisms and mechanisms of actions. Nitrocompounds can be metabolized by ruminal microbials, such as *Denitrobacterium detoxificans*, or alcohol dehydrogenase in the liver. Moreover, it has been found that nitrocompounds are capable of inhibiting pathogens, parasites, methane and ammonia production; however, overdose of nitrocompounds could cause methemoglobinemia or interfere with energy production in mitochondria by inhibiting succinate dehydrogenase.

## 1. Introduction

Nitrocompounds are derivatives of hydrocarbons, alcohols, fatty acids, and esters which contain one or more nitro functional groups (-NO2) [1]. Short chain aliphatic nitrocompounds, such as unitary alphatic nitroalkanes, aliphatic nitroalcohols, and aliphatic nitroacids, have been widely used in the chemical industry because they are readily accessible and stable for syntheses of various organic compounds [2]. Most of the aliphatic nitrocompounds are not technically produced from biological sources, but 3-nitropropanol (3NPOH) and 3-nitropropionic acid (3NPA) can be extracted from *Astragalus*, *Coronilla*, and *Indigofera* genera of the *Leguminosae* family [3]. The most common sources of nitrocompounds in nature are glucose esters of nitropropionic acid and glycoside of nitro-propanol, 3-nitro-1-propyl-beta-D-glucopyranoside, collectively known as miserotoxin. This toxin was first isolated from *Astragalus oblongifolius* [4,5]. Moreover, 3NPA and 3NPOH are observed in fungi, such as *Penicillium* spp. and *Aspergillus* spp., as well as kernel of the fruit of the karaka tree (*Corynocarpus laevigatus*) [6]. Understanding the properties and functions of nitrocompounds may provide us with novel insights and strategies for future application of nitrocompounds in the animal industry. Therefore, we have reviewed the literature and highlight how alphatic nitrocompounds, including 3-nitropropanol, 2-nitro-1-propanol, nitroethane, and 2-nitroethanol, impact on animal production.

## 2. Inhibition of Pathogenic Bacteria, Ammonia and Methane Production

Previous studies have reported that nitrocompunds exhibit broad-spectrum antimicrobial activity both in vitro and in vivo [7,8,9,10,11]. The effects of nitrocompounds on pathogen inhibition have been reviewed and listed in Table 1. Jung et al. [12] and Dimitrijevic et al. [13] indicated that *Enterococcus faecalis* and *Listeria monocytogenes* were reduced in the medium containing 10 mM 2-nitro-1-propanol (2NPOH), whereas *Salmonella Typhimurium* and *Escherichia coli* were significantly inhibited by 2.5 mM 2NPOH. An unpublished test conducted in our lab also showed that 2NPOH (4 and 8 mM) and 2-nitroethanol (2NEOH) (8 mM) significantly inhibited growth of *Clostridium perfringens*. Moreover, 2NPOH, 2NEOH, nitroethane (NE), and 2-nitro-methyl-propionate have been reported to reduce *Campylobacter jejuni* and *Campylobacter coli* in culture of Bolton broth at pH 8.2, whereas 2NPOH are more capable inhibiting *Listeria monocytogenes* than 2NEOH and NE [13,14]. Additionally, Kim et al. [15] reported that 2NEOH, 2NPOH, and 3NPA have the potential to reduce uric acid-utilizing microorganisms isolated from poultry manure. The study also suggested that nitrocompounds had superior inhibitory effects compared to their acid and alcohol counterparts [15]. Furthermore, 2NPOH reduced *Listeria monocytogenes*, *Salmonella enterica* serovar Enteritidis, *Escherichia coli*, *Staphylococcus aureus*, and *Bacillus cereus* inoculated on Russian-type salad and corn-flour-based doughs [11,16,17].

On the other hand, previous studies reported the effects of nitrocompounds on the microbial community in the animal intestine, manure or ruminal fluid. Jung et al. [8] demonstrated that a bird gavaged with 13, 65 and 130 mg 2NPOH inhibited *Salmonella Typhimurium* and regulated volatile fatty acids in the cecal content, whereas broilers fed with 16.7 ppm 2NPOH significantly reduced ammonia nitrogen in feces [20]. It is suggested that nitrocompounds reduce ammonia production by inhibiting uric acid-utilizing microorganisms in animal manure [10,20]. Early work by Kim et al. [10] confirmed that 100 mM 2NPOH and 3NPA significantly suppressed uric acid-utilizing microorganisms isolated from poultry feces. Furthermore, Ruiz-Barrera et al. [19] observed that NE reduced *Escherichia coli* and total coliforms after incubation of layer hen manure for 24 h, whereas 2NEOH and 2NPOH reduced *Salmonella Typhimurium* in feces of 6-month-old poultry litter and manure collected from mature sows [18]. Additionally, nitrocompounds regulated immune responses in laying hens challenged with *Salmonoella* [7]. The previous study reported that *Salmonella* challenge increased gene expression of interferon-γ, interleukin-1B, and Toll-like receptor-4 in the ileum of laying hens, but 2NPOH downregulated these cytokines and numerically reduced *Salmonella* in the ceca [7].

Nitrocompounds not only inhibited pathogens and reduced ammonia production in poultry, but also decreased skatole levels in swine manure [21], as well as methane production in ruminants [22]. Zhang et al. [1] summarized the roles of nitrocompounds as methanogenic inhibitors in ruminant animals. Nitrocompounds were first evaluated in an in vitro study [23], indicating that methane production was inhibited by 2NPOH, NE, 2NEOH in the ruminal fluid collected from Holstein-Friesian cows. These results were in agreement with another in vitro study which suggested that NE, 2NPOH, and 2NEOH enhanced volatile fatty acids production and reduced methane formation in the broiler cecal content after 24 h inoculation [24]. Moreover, several in vivo studies have been conducted to confirm the methane-inhibiting ability of selected nitrocompounds. Anderson et al. [9] demonstrated that daily administration of 2NEOH and 2NPOH reduced methane production in mature ewes, whereas Gutierrez-Banuelos et al. [25] reported that NE inhibited methane-producing ability in the rumen and feces of steers. Furthermore, methane emissions and the ratio of acetate to propionate were linearly reduced in response to the increase in NE supplementation. To access the effect of NE on methanogenesis, the same research group conducted an in vitro test. The study showed that NE increased nitro-metabolizing bacteria, reduced methane production, but did not accumulate hydrogen levels in the ruminal fluid [26]. Apart from alphatic nitroalkanes, nitroalcohols, and nitroacids, another nitrocompound has been studied recently. It has been reported that 3-nitrooxypropanol (3NOP) also has methane-inhibitory effects in ruminants [22,27,28]. In addition, dairy cows could produce less methane production for 10 additional weeks after withdrawal of 3NOP [29]; thus, it suggested that 3NOP might be a potential feed additive acting as a methane inhibitor in ruminant animals [1].

## 3. Inhibition of *Eimeria* spp.

Interestingly, nitrocompounds act like monensin, in terms of both inhibiting methane production and suppressing parasite colonization in ruminants and chickens [23,30,31,32,33,34,35]. Teng et al. [32] demonstrated that 0.5 mg/mL of monensin and 0.5 mM 2NPOH and 2NEOH significantly inhibited development of sporozoites in the Madin-Darby bovine kidney cells. Moreover, dietary supplementation of 200 ppm 2NPOH reduced cecal lesion scores, as well as improved digestibility of energy in the birds challenged with *Eimeria* spp. [32]. However, 2NPOH did not improve intestinal permeability in a recent experiment [35]. In the ruminants, NE and monensin exhibit similar effects on inhibition of methane production [26,33]. Furthermore, monensin could further regulate butyrate formation, whereas NE did not show significant effects on production of ruminal volatile fatty acids [26]. A recent study was conducted to compare the effects of monensin and NE on digestibility and growth performance of lambs. The authors demonstrated that both monensin and NE did not improve digestibility of crude protein, organic matter, neutral detergent fiber, and acid detergent fiber [33]. However, lamb fed with NE had higher average daily gain and better feed conversion rate compared to the group administrated with monensin [33].

## 4. Toxicity of Nitrocompounds

Even though nitrocompounds induce several positive outcomes on inhibiting pathogens as well as reducing methane and ammonia production in the gastrointestinal tracts, the toxicity of these organic compounds has caused consumers’ caution. Previous in vitro studies have reported that 0.4 to 3 mM is the range of cytotoxic threshold of various testing cells following exposure to nitrocompounds [32,36,37]. It should be noted that the toxicity thresholds of cells are much lower than the thresholds of pathogens (from 4 to 50 mM), indicating that animal cells are more vulnerable than pathogens to nitrocompounds.

Ingestion of *Astragalus* spp. may cause livestock poisoning in ruminant and monogastric animals [38]. Moreover, several reviews have concluded that miserotoxin of *Astragalus* spp. is less toxic to ruminants than nonruminants after oral administration [3,39]. Miserotoxin (3-nitro-1-propyl-beta-D-glucopyranoside) was isolated and characterized from *Astragalus* spp. by Stermitz et al. [5]. The concentrations of miserotoxin in *Astragalus* spp. vary from 2 to 6% [40]. Miserotoxin is relatively innocuous to animals compared to the pure nitrocompounds, such as 3NPA or 3NPOH. A previous study reported that the LD50 of miserotoxin to rats was greater than 2.5 g/kg, whereas the LD50 of 3NPOH was 77 mg/kg [41]. The symptoms of toxicity caused by miserotoxin or nitrocompounds are similar to methemoglobinemia, including depression of feed intake, a tendency to fall down, difficulty in breathing and head extension [3]. These observed reactions in animals are also classic symptoms of nitrite poisoning. However, nitrocompounds do not cause lethal levels of methemoglobinemia as nitrite does [3]. The toxicity of 3NPA and 3NPOH in humans and animals has been reviewed by [4,6]. The 3NPA does not exhibit chronic toxicity; furthermore, the acute toxicity of LD50 dose of 3NPA is between 60 to 120 mg/kg (oral challenge). Burdock et al. [6] also concluded that the acceptable daily intake of 3NPA should not be above 25 mg/kg/day for human.

Toxicity levels of nitroalkanes and nitroalcohols have been concluded in a previous review article. Smith et al. [39] indicated that the acute LD50 values for mice following intraperitoneal injection of nitromethane, NE, 1-nitropropane, 2-nitropropane, and 2NEOH were 110, 310, 250, 800, and 2100 mg/kg body weight, respectively. Moreover, rats under inhalation exposure to NE at 100 or 200 ppm for 2 years had no significant effects on body weights, hematology, nonneoplastic, and neoplastic pathology [42]. An early study also demonstrated that supplementation of less toxic nitrocompounds, such as NE, was capable to prevent *Astragalus* spp. poisoning in ruminants [43].

Less is known regarding toxicity of dietary supplementation of synthetic pure nitrocompounds on economic animals. Previous studies have not indicated any adverse effect of nitrocompounds on performance of laying hens and ovine [7,9]. However, Jung et al. [8] reported that 6-day-old broiler chickens gavaged with a single dose of 130 mg 2NPOH caused 30% mortalities, whereas 13 mg 2NPOH showed no apparent adverse effects. Moreover, dietary supplementation of 33 and 100 ppm 2NPOH and 2NEOH had no impacts on growth performance of young broiler chickens, but 200 ppm 2NPOH and 2NEOH addition resulted in decrease in body weight [20,32]. In summary, toxicity of nitrocompounds is diverse and is influenced by various factors, including animal species, ages, and types and doses of nitrocompounds. Little evidence of chronic toxicity caused by nitrocompounds was reported in previous studies; thus, further investigation is needed before the application of nitrocompounds in animal production.

## 5. Metabolism of Nitrocompounds

**Figure 1:**
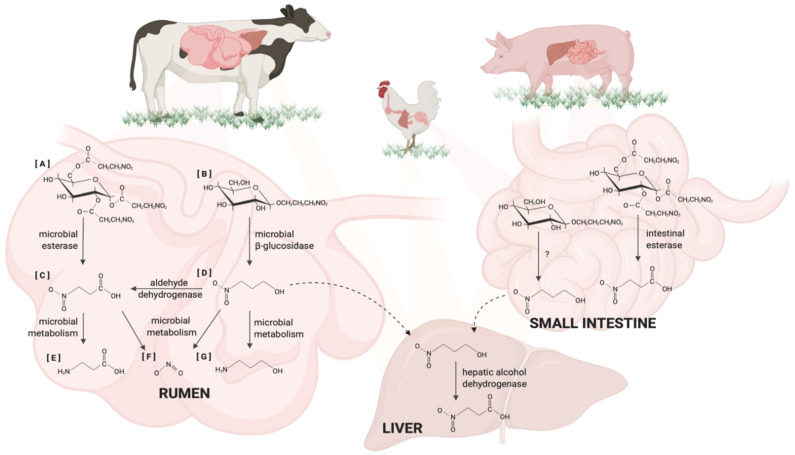
Metabolism of natural sources of nitrocompounds in ruminants and non-ruminants. (**A**) Glucose esters of 3-nitropropionic acid; (**B**) glycoside of 3-nitro-propanol (3-nitro-1-propyl-beta-D-glucopyranoside); (**C**) 3-nitropropionic acid; (**D**) 3-nitropropanol; (**E**) 3-aminopropionic acid (β-alanine); (**F**) nitrite; (**G**) 3-aminopropanol.

The metabolism of natural sources of nitrocompounds is illustrated in Figure 1. In ruminants, glycoside of nitro-propanol and glucose esters of nitropropionic acid are hydrolyzed by microbial β-glucosidase and esterase, respectively, in the rumen. The rate of hydrolyzation is estimated at 0.75 g mol/mL/h in ruminal fluid [44]. After liberating free 3NPA and 3NPOH, ruminal microbials rapidly metabolize 3NPOH to 3NPA, indicating that these nitrocompounds are equally toxic to animals [44,45]. Apart from metabolizing to 3NPA, 3NPOH is also oxidized to 3-aminopropanol, whereas 3NPA is further metabolized to 3-aminopropionic acid (β-alanine) in the rumen [46]. A previous study indicated that the metabolism of 3NPA was faster than 3NPOH, and the disappearance of 3NPOH was proceeded at a faster rate than that of 2NPOH [44]. It has been reported that the efficiency of ruminal metabolism was influenced by dietary treatments, such as supplementation of NE [43,47]. Dietary protein also contributed to the rate of microbial detoxification [43]. A recent study demonstrated that ruminal microbials could cleave 3NPOH and 3NPA to nitrite [48], and the nitrate will further be degraded to ammonia by rumen microorganisms [49]. However, nitrocompounds are not only metabolized to their respective amines, nitrite, and ammonia, but are also directly absorbed by reticulo-rumen in both sheep and cattle [50,51]. If the 3NPOH was not metabolized to 3-aminopropanol, 3NPA, or nitrate in the rumen, it might be further metabolized to 3NPA in the liver [52]. 

Unlike ruminants, monogastric animals, such as pigs and chickens, are not able to secrete β-glucosidase; thus, they absorb miserotoxin in the upper gastrointestinal tract [4] (Figure 1). Though little is known regarding how non-ruminants hydrolyze miserotoxin to liberate free 3NPOH after absorption, previous studies indicated that free 3NPOH is metabolized to 3NPA by aldehyde dehydrogenase and hepatic alcohol dehydrogenase [52,53]. Moreover, monogastric animals are able to metabolize glucose esters of nitropropionic acid by tissue esterase [4].

The metabolism of NE, 2NEOH, and 2NPOH in animals might not share the same pathway as 3NPOH and 3NPA. It has been suggested that NE is transformed to acetaldehyde and nitrite in animals [39]. The acetaldehyde might be oxidized to acetate by acetaldehyde dehydrogenase and further enter into tricarboxylic acid (TCA) cycles, but nitrite is critical to cause acute poisoning [39,54]. Zhang et al. [48] reported that 90% of NE could be degraded by microorganisms, whereas only 75% of 2NEOH and 60% of 2NPOH were metabolized in the ruminal fluid. Moreover, NE, 2NEOH, and 2NPOH might be degraded to ethylamine, amino-ethanol, and 2-amino-1-propnol, respectively, and these intermediates might be further metabolized to nitrite and ammonia by ruminal microbials. The authors also suggested that NE and 2NPOH produced more ammonia compared to 2NEOH [48].

Several ruminal bacteria are able to degrade nitrocompounds and metabolize nitrite anaerobically, including *Megasphaera* spp., *Coprococcus* spp., *Ramibacterium* spp., and *Selenomonas* spp. [49]. Moreover, a new group of anaerobic bacteria, *Denitrobacterium detoxificans*, was identified by Anderson et al. [55]. The author demonstrated that growth of *Denitrobacterium detoxificans* was supported by 3NPA, 3NPOH, 2NPOH, NE, and 2NEOH as electron acceptors, whereas hydrogen and formate served as electron donors. Furthermore, *Clostridium* spp. also has similar effects on the reduction of aliphatic nitrocompounds by hydrogenase and ferredoxin [56].

## 6. Mechanisms of Actions of Nitrocompounds

**Figure 2:**
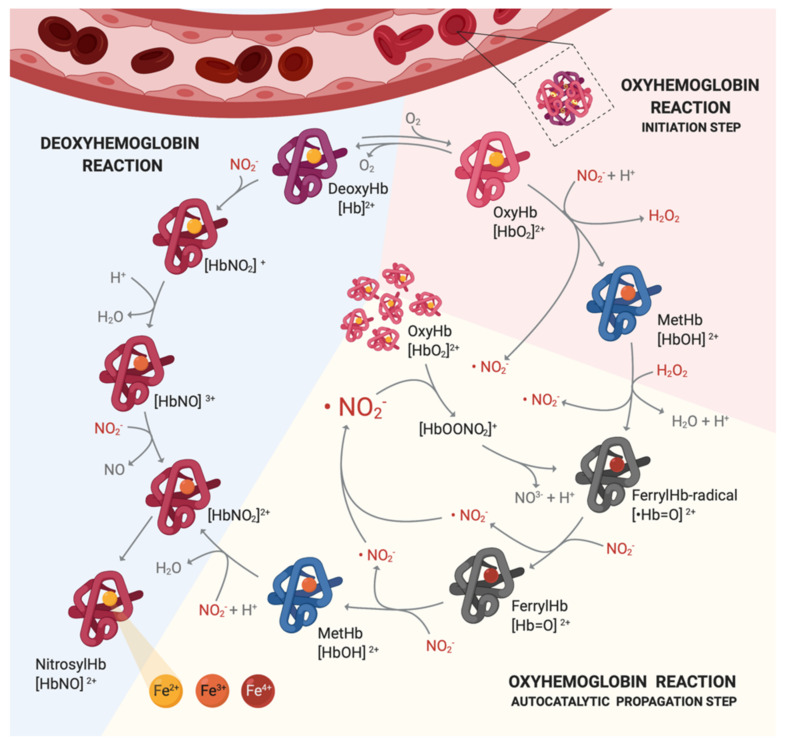
Mechanisms of actions of nitrite poisoning caused by nitrocompounds administration. Once nitrocompounds are metabolized to nitrite in the gastrointestinal tracts in animals, nitrite will interact with both oxyhemoglobin and deoxyhemoglobin, leading to failure of oxygen transportation. Nitrite could further initiate an autocatalytic propagation that keep oxidizing oxyhemoglobin to methemoglobin and end up with the formation of nitrosyl hemoglobin.

Three mechanisms of actions of nitrocompounds have been proposed in previous studies, explaining how nitrocompounds cause toxicity to animals and how nitrocompounds inhibit pathogens and methanogenesis [55,57,58,59,60]. The most common toxicity of nitrocompounds is associated with nitrite poisoning shown in Figure 2. After being reduced by ruminal microbial, nitrite acts as a strong reductant in the circulation which rapidly reduces ferrous (Fe^2+)^ iron in oxyhemoglobin (oxyHb) to ferric (Fe^3+^) state also known as methemoglobin (metHb). The reaction between nitrate and oxyHb not only generates methemoglobin, but also produces hydrogen peroxide and nitrate. The hydrogen peroxide will initiate an autocatalytic propagation with metHb, forming a ferrylhemoglobin (ferrylHb)-radical. The ferrylHb-radical reduces back to metHb by generating two molecules of nitrogen dioxide from nitrite. The nitrogen dioxide can further oxidize oxyHb to ferrylHb-radical, leading to the unstoppable autocatalytic propagation. As the propagation will not be terminated until most of the nitrite in the circulation system is consumed [61], the serial reactions elevate metHb precipitously. Moreover, deoxyhemoglobin also reacts with nitrite, forming nitrosyl hemoglobin as the end product [62]. Both MetHb and Nitrosyl hemoglobin are incapable of carrying oxygen; thus, animals fed high levels of nitrocompounds or nitrite will fail to transport oxygen to tissue and result in death due to severe hypoxia [57].

It is speculated that the more nitrite generated from nitrocompounds, the stronger toxicity that might be observed. As it is discussed above, in the ruminants, nitrocompounds are degraded to various organic compounds by ruminal microorganisms, whereas monogastric animals only produce nitrite as the final product. If animals were fed the same amount of nitrocompounds, ruminants could generate less nitrite than non-ruminants do; thus, previous studies have concluded that miserotoxin is less toxic to ruminants than non-ruminants [39]. 

**Figure 3:**
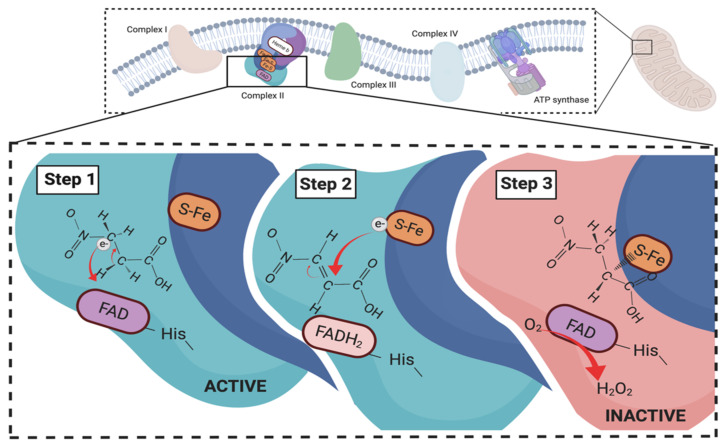
Inhibition of succinate dehydrogenase in electron transport chain by 3-nitropropionic acid (3NPA). 3NPA has a similar chemical structure as succinic acids which make it possible to attach on succinate dehydrogenase and irreversibly inactive the enzyme, causing failure of electron transportation in mitochondria.

On the other hand, 3NPA could inhibit succinate dehydrogenase (SDH) and manipulates energy production of cells (Figure 3). SDH plays important roles in both TCA cycle and respiratory complex II, an enzyme involved in the electron transport chain [63]. SDH regulates oxidation of succinate to fumarate and the reduction of quinone to quinol in the membrane [63]. Hylin et al. [64] first reported the effects of 3NPA on SDH in the heart muscle of rat. It was proposed that 3NPA can act as a suicide inhibitor of SDH because chemical structure of 3NPA is similar to succinic acid, the substrate of SDH [58]. Coles et al. [59] further summarized how 3NPA inactivates the enzyme. In the initiating step, 3NPA is metabolized to 3-nitroacrylate, following with two electrons transferring to the flavin and generating reduced flavin adenine dinucleotide (FADH2) on the enzymes. The nucleophilic of a thiol group is later added to the double bound of 3-nitroacrylate, formatting a thioether on the SDH. Even though FADH2 can be oxidized by respiratory chain, the 3-nitroacrylate is not able to release from the enzyme anymore; thus, the effect of 3NPA on SDH is considered as an irreversible reaction. Moreover, a previous study reported that nitrocompounds could inhibit formate dehydrogenase, formate hydrogen lyase, and hydrogenase activity [60]. As SDH, formate dehydrogenase, and formate hydrogen lyase play important roles in energy metabolism in both eukaryotes and prokaryotes, it is concluded that nitrocompounds might impede energy production in pathogens and parasites by suppressing metabolism of formate and hydrogen as well as inhibiting the SDH involved in complex II and TCA cycle [65,66,67].

**Figure 4:**
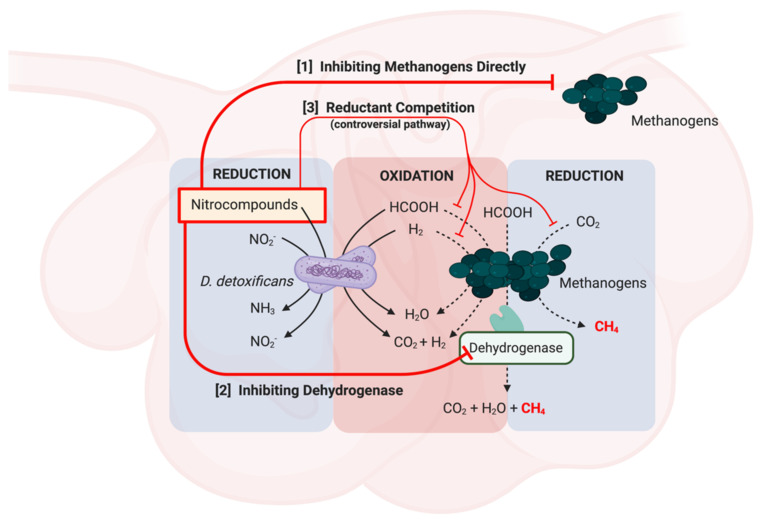
Possible mechanisms of actions of nitrocompounds withhold methanogenesis in ruminants. Nitrocompounds might directly inhibit methanogens [pathway 1] or suppress dehydrogenase which is an enzyme metabolizing formate to methane [pathway 2]. Metabolism of Nitrocompounds by D. detoxificans consumes reductant in the rumen, such as hydrogen and formate. Nitrocompounds might compete these reductants with carbon dioxides, indirectly reducing methane production. [pathway 3] Reductant competition is a possible mechanism of action of nitrocompounds, but it might not play the main role on inhibition of methanogenesis in ruminants.

The presence of *Denitrobacterium detoxificans* and the inhibition of formate dehydrogenase by nitrocompounds are the main mechanisms of actions that reduced methane production in the ruminants. *D. detoxificans* processes nitrocompounds metabolizing activity and causes the reduction of nitrite, nitroalkanes and nitroalcohols with oxidation of hydrogen and formate (Figure 4). In the reaction, hydrogen and formate are oxidized to water, carbon dioxide and hydrogen, whereas nitrite and nitrocompounds are reduced to ammonia and nitrite, respectively [68]. Zhang et al. [1] further concluded that nitrocompounds act as alternative electron acceptors, diverting the flow of reducing equivalent away from methanogenesis (Figure 4, [pathway 3]). On the contrary, other studies indicated that the inhibition of methane production was independent to the presence of *D. detoxificans* and the loss of reducing equivalents by the reduction of nitrocompounds [23,69]. Though competing reductants might not be the main mechanisms of actions of nitrocompounds, it should be noted that metabolizing nitrocompounds by *D. detoxificans*, indeed, spares the reducing equivalents from the production of methane [68].

It has been proposed that nitrocompounds reduce methane production by inhibiting methanogens directly or suppressing ability of formate dehydrogenase [60] (Figure 4, [pathway 1] and [pathway 2]). The ruminal methanogens are capable of metabolizing formate to carbon dioxide and hydrogen, which are rapidly oxidized to methane [70]. Additionally, some methanogens could degrade formate to carbon dioxides, water, and methane directly via formate dehydrogenase [71]. Approximately 18% of ruminal methane was formed from formate rather than carbon dioxide [72]; thus, inhibiting dehydrogenase for formate oxidation is a potential mechanism of action of nitrocompounds in withholding methanogenesis in the rumen.

## 7. Conclusions

Conjugates of 3NPOH and 3NPA in forages, and various synthetic nitrocompounds have been reviewed in the context of their effects on the inhibition of foodborne pathogens, parasites, methane, and ammonia production in economic animals. The toxicity, metabolism, and mechanisms of actions have been discussed in the current review to conclude the advantages and disadvantages of application of nitrocompounds in animal production. Previous studies have elucidated the properties of 3NPA and 3NPOH comprehensively because they are the only nitrocompounds observed from natural sources so far. It has been demonstrated that the toxicity of 3NPA and 3NPOH is associated with nitrite poisoning and inactivation of SDH. Nevertheless, little is known regarding the mode of actions and toxicity of 2NPOH, NE, and 2NEOH. Even though the effects of short-chain nitrocompounds on broiler chicken, laying hen, cattle, lamb and swine have been studied for decades, further research is needed to determine a range of safe dosages in order to use nitrocompounds as a novel strategy for the control of pathogens in animal production.

## Figures and Tables

**Table 1:** Summary of Antimicrobial ability of nitrocompounds in vitro and in vivo.

Nitrocompound	Dosage	Unit	Pathogens Inhibition	Reference
			*In vitro*	
2NEOH	10, 20	mM	*Campylobacter coli*	[14]
	10, 20	mM	*Campylobacter jejuni*	[14]
	8	mM	*Clostridium perfringens*	Unpublished data
	15	mM	*Listeria monocytogenes* strain 18	[13]
	50	mM	uric acid-utilizing microorganisms	[15]
2NMP	10, 20	mM	*Campylobacter jejuni*	[14]
2NPOH	5	%	*Bacillus cereus* ^1^	[11]
	10, 20	mM	*Campylobacter coli*	[14]
	10, 20	mM	*Campylobacter jejuni*	[14]
	4, 8	mM	*Clostridium perfringens*	Unpublished data
	10	mM	*Enterococcus faecalis*	[12]
	2.5, 5, 10	mM	*Escherichia coli*	[12]
	0.5, 2, 5	%	*Escherichia coli* ^1^	[11]
	10, 15	mM	*Listeria monocytogenes* strain 18	[13]
	50	mM	*Listeria monocytogenes* ^2^	[17]
	0.5, 2, 5	%	*Salmonella enterica* serovar Enteritidis ^1^	[11]
	2.5, 5, 10	mM	*Salmonella Typhimurium*	[12]
	0.5, 2, 5	%	*Staphylococcus aureus* ^1^	[11]
	50	mM	uric acid-utilizing microorganisms	[15]
3NPA	50	mM	uric acid-utilizing microorganisms	[15]
NE	10, 20	mM	*Campylobacter coli*	[14]
	10, 20	mM	*Campylobacter jejuni*	[14]
	15	mM	*Listeria monocytogenes* strain 18	[13]
			In vivo/feces incubation	
2NEOH	20	mM	*Escherichia coli*	[18]
	20	mM	*Escherichia coli*	[18]
	44	mM	*Salmonella Typhimurium*	[19]
	13, 65, 130	mg/bird	*Salmonella Typhimurium* ^2^	[8]
	44	mM	*Salmonella Typhimurium*	[19]
	20	mM	*Salmonella Typhimurium*	[18]
	100	mM	uric acid-utilizing microorganisms	[10]
3NPA	100	mM	uric acid-utilizing microorganisms	[10]
Ethyl-nitroacetate	44	mM	*Salmonella Typhimurium*	[19]
NE	12	mM	*Escherichia coli*	[19]
	44	mM	*Salmonella Typhimurium*	[19]
	20	mM	*Salmonella Typhimurium*	[18]
	12	mM	Total Coliforms	[19]

2NEOH, 2-Nitroethanol; 2NMP, 2-Nitro-methyl-porprionate; 2NPOH, 2-Nitro-1-propanol; 3NPA, 3-Nitropropionic acid; NE, Nitroethane. ^1^ Inoculated on corn flour-based doughs; ^2^ Inoculated on Russian-type salad.

## Data Availability

No new data were created or analyzed in this study. Data sharing is not applicable to this article.
